# Competencies of Nurse Managers as Predictors of Staff Nurses’ Job Satisfaction and Turnover Intention

**DOI:** 10.3390/ijerph191811461

**Published:** 2022-09-12

**Authors:** Pin-Pin Choi, Wai-Man Lee, Suet-Shan Wong, Mei-Ha Tiu

**Affiliations:** 1 School of Nursing and Health Studies, Hong Kong Metropolitan University, Hong Kong; 2 School of Nursing, St. Teresa’s Hospital, Hong Kong

**Keywords:** management, professional roles, survey, workforce

## Abstract

Nurse managers have played an integral role in stabilizing the nursing work environment and workforce in the face of the COVID-19 pandemic, yet the competencies required for such a feat are largely unknown. This study was conducted during the pandemic to identify the specific domains of nurse manager competencies that associate with nurse outcomes. A cross-sectional survey was conducted on a convenience sample of 698 staff nurses to measure the perceived competence of their nurse managers and their job satisfaction and turnover intention levels. The overall perceived nurse manager competency level in our sample was 3.15 out of 5 (SD = 0.859). The findings indicated that 34.3% of nurses were dissatisfied with their current jobs, and 36.3% of nurses were considering leaving their current workplace. Regression analyses identified “Team Communication and Collaboration” (β = 0.289; *p* = 0.002), “Staff Advocacy and Development” (β = 0.229; *p* = 0.019), and “Quality Monitoring and Pursuance” (β = 0.213; *p* = 0.031) as significant predictors of staff nurses’ job satisfaction and “Staff Advocacy and Development” (β = −0.347; *p* < 0.000) and “Team Communication and Collaboration” (β = −0.243; *p* = 0.012) as significant predictors of nurses’ turnover intention. The findings of the study have implications for the future recruitment, training, and performance evaluation of nurse managers.

## 1. Introduction

### 1.1. Challenges in Modern Healthcare

Modern healthcare continues to face increasing challenges due to growing aging populations and the escalating demands of care recipients for quality care [1]. The boom in the medical sciences and technology has added to these challenges by contributing to new knowledge on disease management, leading to constant changes in healthcare policies and care delivery processes [1]. The outbreak of the coronavirus disease 2019 (COVID-19) has further intensified the complexity of the healthcare environment. Healthcare workers have developed psychological distress and burnout due to overwhelming patient loads, inadequate manpower and time off, and separation from family members during the pandemic [2]. The same applies to nurses, who make up the largest proportion of the healthcare workforce and provide round-the-clock care to patients. The pandemic has increased their job demands, leading to feelings of loss of control, burnout, and moral distress [3]. The International Council of Nurses (ICN) published a report entitled “The global nursing workforce and the COVID-19 pandemic” in January 2022 to raise an urgent appeal about the sustainability of the nursing workforce [4]. The nurse understaffing phenomenon is expected to persist for some years, and the ICN has prompted health service providers to develop effective strategies to mitigate the impact of the pandemic on the phenomenon [4].

### 1.2. The Pivotal Role of First-Line Nurse Managers

First-line nurse managers play a central role in confronting the challenges in healthcare as they are responsible for overseeing the operation of individual work units, and for providing direct supervision and work support to frontline nurses [1,5,6]. Their competencies, namely the specific attributes and behaviors crucial for effective performance, were extensively studied. Previous research on nurse manager competencies focused on examining their leadership styles and management practices, which have been cited as integral to staff satisfaction and retention [5,7,8,9,10]. Cummings and colleagues [7] conducted a systematic review and found a strong linkage between leadership and management behaviors and various nurse outcomes, including individual productivity and effectiveness, staff health and well-being, and staff satisfaction and retention. Many scholars further pinpointed a lack of attention paid by researchers, hospital administrators, and policy makers to the importance of unit-level management, and they have called for more systematic studies to examine the competencies required of nurse managers and the management skills and practices that determine nurse outcomes [5,11,12].

During the COVID-19 pandemic, there has been increasing scholarly interest in examining workforce issues in nursing. Researchers are cognizant of the exacerbating effect of the pandemic on nurse shortages and turnover. Most studies focused on the work-related outcomes and experiences of nurses, capturing their perceived stress, satisfaction, support, intent to stay/leave, and quality of care [13,14], as well as their lived experiences of caring for patients during the pandemic [15,16], through survey and qualitative approaches. There is also growing evidence confirming the relationship between the quality of the practice environment and nurse outcomes, with management support being one of the dimensions underlying the construct of the practice environment [17,18]. However, very few studies have examined the management behaviors that support nursing work during the pandemic. Researchers examined the associations between nurse outcomes and their practice environment, and noted that “dissatisfaction with management” is the top-ranked factor leading to the intention of nurses to leave their workplace [19]. They emphasized the role of nurse managers in rebuilding the nursing work environment and retaining nurses and stressed that there is an urgent need to further explore issues related to the competencies of nurse managers and nurse outcomes in light of the pandemic [19,20].

### 1.3. Background of This Study

This study was conducted during the COVID-19 pandemic and was developed based on a phenomenological study that delineated the perceptions of first-line nurse managers and staff nurses about what constitutes the managerial effectiveness of nurse managers [21]. The study generated a list of narrative descriptions that captured the competencies required of nurse managers and laid the foundation for this study. According to the extant literature, nurse manager competencies are closely associated with nurse outcomes [7,8]. This study set out to test this hypothesis and to shed light on the competencies of nurse managers as perceived by staff nurses and nurse satisfaction and turnover during the pandemic.

## 2. Aim

The aim of this study was to identify the specific domains of nurse manager competency that associate with nurse outcomes, namely the job satisfaction and turnover intention of staff nurses.

## 3. Methods

### 3.1. Design

A cross-sectional survey design using online questionnaires was employed in this study.

### 3.2. Sampling and Data Collection

Convenience and snowball sampling approaches were adopted. Potential respondents were invited to complete an electronic survey in a structured Google form, which was disseminated to the hospital administrators of both public and private hospitals in Hong Kong as well as through the research team’s personal networks. Eligible respondents were those who were full-time staff nurses currently practicing in hospital settings in Hong Kong. The Google form contained an information sheet, and relevant questions to screen for eligibility of the respondents. Only one response per IP address was allowed. Nurse managers and administrators were excluded because they were not involved in direct patient care. Data collection was conducted from May to July 2022. The survey was distributed to 824 potential respondents, and the response rate was 84.7% (*N* = 698). Among them, 688 questionnaires were filled out completely and were used for the final analysis. The sample size of this study was estimated using G*Power v.3.1.9.4 (USA) for a multiple linear regression analysis, using a medium effect size of 0.15, an α error probability of 0.05, a power of 0.95, and five predictors. The sample size required was 138, and the sample size attained in this study was sufficient for statistical analyses.

Ethical approval was obtained from the institution with which the corresponding author was affiliated prior to the commencement of the study (Reference no: HE/RGC/2019-31). Respondents were provided with written information on the aim and process of the study, and on their right to confidentiality and to withdraw from the study. The survey was anonymous, so no personal identifying information was collected from the respondents. Informed consent regarding the voluntary participation of the respondents was indicated by the return of their questionnaires.

### 3.3. Measures

Data collection was performed using an instrument developed from a phenomenological study that involved individual interviews with both nurse managers and staff nurses to elicit their perspectives on the competencies required of nurse managers [21]. Nurse manager competencies refer to the attributes and behaviors essential for managerial competence [21]. A total of 63 descriptive expressions were generated from the verbatim transcripts, which were then content validated by four expert panels who were nurse managers and/or administrators with at least 15 years of experience in nursing leadership and management. Content validation involves examining whether an instrument is made up of an appropriate sample of items for measuring the construct under study [22]. All panel members consensually agreed that the items are appropriate for measuring the essential competencies of nurse managers. Minor modifications were made to two items to improve clarity, and no items were added or deleted from the instrument. The scale-level content validity index of the instrument was 0.94, which indicated a high level of relevance and clarity [22].

The instrument was named the *Nurse Manager Competency Scale*, and its psychometric properties were further evaluated by 970 staff nurses recruited from February to May 2022. The sample size was estimated based on a 1:10 item-to-response ratio [23], and sampling and data collection were conducted using the same approaches adopted in this study. Exploratory factor analysis (EFA) was adopted to examine the construct validity of the scale, which refers to the extent to which the instrument can represent a theoretical construct [24]. Principal axis factoring with varimax rotation was chosen as it allows for the existence of correlations among domains [25]. The Kaiser–Meyer–Olkin (KMO) test and Bartlett’s test for sphericity were performed before the EFA. The number of factors extracted was conducted according to the Kaiser–Guttman rule, taking into consideration the following criteria: (1) eigenvalues of 1.0 or above; (2) scree plots; (3) items with salient factor loadings of ≥0.40; and (4) the meaning of the items [26]. The results yielded a five-factor structure that accounted for 58.3% of the total variance. Three items were removed as they were noted to be redundant and were with low factor loadings (<0.40). Internal consistency coefficients were computed using the Cronbach’s alpha measure to indicate the degree to which the items measure the same trait [24]. The internal consistency coefficient of the overall scale was 0.96, reflecting that the instrument is reliable [27].

Three main variables were examined in this study, namely the nurse manager competencies, and job satisfaction and turnover intention of staff nurses. The resulting *Nurse Manager Competency Scale* adopted in this study was made up of 60 items that captured five domains of *nurse manager competencies*: (1) Staff Advocacy and Development [12 items], which measured the competencies in advocating for staff interests and developing staff potential; (2) Team Communication and Collaboration [15 items], which measured the competencies in building cohesive teams through fostering team communication and collaboration; (3) Change and Resource Management [10 items], which measured the competencies in facilitating and embracing changes and ensuring optimal resource allocation; (4) Quality Monitoring and Pursuance [11 items], which measured the competencies in safeguarding the quality of care and cultivating a quality culture in the work unit; and (5) Personal Mastery [12 items], which measured the competencies in knowing oneself, his or her own internal attributes, purpose and vision [21]. Respondents were asked to evaluate the competencies of their nurse managers by using a 5-point Likert scale, ranging from 1 (strongly disagree) to 5 (strongly agree). Average scores were computed for each subscale and the overall scale, with higher scores denoting a more positive perception towards the competencies of their nurse managers and vice versa. For nurse outcomes, in line with the extant literature, *job satisfaction* and *turnover intention* were measured using a single-item measure [28,29]. Respondents were asked to indicate their level of satisfaction with their current job, and their intention to leave their current workplace, by using a 5-point Likert scale, ranging from 1 (most dissatisfied/least intended to leave) to 5 (most satisfied/most intended to leave). For the job satisfaction measure, a higher score indicates a higher level of job satisfaction and vice versa, and it is postulated as positively related to the nurse manager competencies. For the turnover intention measure, a higher score indicates a higher level of intention to leave the current workplace, and vice versa, and it is postulated as negatively related to the nurse manager competencies. Demographic and work-related information, including gender, age, rank, years of work experience, and type of hospital and work setting were also collected.

Information regarding the instrument used in this study, and its psychometric properties (including the factor analysis results and Cronbach’s alpha values of each domain) is presented in Table 1. The questionnaire was written in Chinese, and was translated into English for reporting purposes. Forward and backward translations were conducted by two research team members, and validation was carried out by a qualified language editor.

### 3.4. Data Analysis

Data were analyzed using SPSS version 26.0 software (Armonk, NY, USA: IBM Corporation). Demographic and work-related data, the perceived nurse manager competencies, and nurse outcome variables (namely job satisfaction and turnover intention) were summarized using descriptive statistics such as frequencies, percentages, means, and standard deviations (SDs). Pearson correlation analyses were performed to examine the associations among all the study variables. Multiple linear regression analyses were conducted to examine the relationships between the five domains of nurse manager competency (predictor variables) and the job satisfaction and turnover intention of frontline nurses (outcome variables). For all the analyses, a *p* value of <0.05 was regarded as statistically significant.

## 4. Results

### 4.1. Demographic and Work-Related Characteristics of the Respondents

The 688 respondents recruited in this study were from 24 hospitals and 88 work units. Among them, the majority were female (83.6%, *n* = 575), and were registered nurses (61.3%; *n* = 422) working in public hospitals in Hong Kong (83.9%; *n* = 577). Around half of them worked in general medical and surgical settings (46.7%; *n* = 321), and approximately 60% of them (*n* = 416) had more than 10 years of work experience. Details of the demographic and work-related characteristics of the respondents are presented in Table 2.

### 4.2. The Level of Nurse Manager Competencies and Nurse Outcomes

Table 3 lists the items along with the respondents’ mean ratings of the nurse manager competencies. The overall mean score on *nurse manager competencies* in our sample was 3.15 out of 5 (SD = 0.859). Among the five domains of nurse manager competencies, the respondents rated “Quality Monitoring and Pursuance” (mean = 3.20; SD = 0.811) the highest, followed by “Personal Mastery” (mean = 3.16; SD = 0.859), “Change and Resource Management” (mean = 3.15; SD = 0.816), “Staff Advocacy and Development” (mean = 3.06; SD = 0.918), and lastly “Team Communication and Collaboration” (mean = 3.05; SD = 0.890). Respondents gave comparatively higher ratings to a few items, including the competencies of nurse managers in “monitoring staff performance to ensure quality of care” (item 45) under the domain of “Quality Monitoring and Pursuance”; “being passionate about work” (item 59); “being clinically knowledgeable and proficient” (item 60) under the domain of “Personal Mastery”; and “responding effectively to sudden increases in resource demand” (item 30) under the domain of “Change and Resource Management”. Respondents gave relatively lower ratings to a number of items, concerning the competencies of nurse managers in “*understanding staff’s unique needs*” (item 1), and “*addressing the needs of staff*” (item 6) under the domain of “Staff Advocacy and Development”, and “*viewing from others’ perspectives*” (item 16), “establishing trusting relationships” (item 17), and “*providing communication platforms*” (item 21) under the domain of “Team Communication and Collaboration”.

For nurse outcomes, the mean *job satisfaction* level was 2.85 out of 5 (SD = 0.875), and the mean *turnover intention* level was 3.16 out of 5 (SD = 0.817). Only one-fourth of respondents (25.0%; *n* = 172) rated themselves as satisfied with their current jobs; more than one-third of respondents were dissatisfied with their current jobs (34.3%, *n* = 236) and were considering leaving their current workplace (36.3%, *n* = 250).

### 4.3. Correlations between Nurse Manager Competencies and Nurse Outcomes

Significant positive associations were noted between job satisfaction and the five domains of nurse manager competencies, and significant negative correlations were noted between turnover intention and the five domains of nurse manager competencies. The strength of the relationships detected was below 0.60, reflecting a moderate relationship between nurse manager competencies and nurse outcomes (Table 4).

### 4.4. Predictors of Nurse Outcomes

Multiple regression analyses were conducted to examine the associations between the five domains of nurse manager competencies and nurse outcomes. The five domains of nurse manager competencies were entered as predictor variables, and the job satisfaction and turnover intention measures were entered as the dependent variables. Correlation analyses revealed weak but significant correlations (*r* ranging from 0.108 to 0.200) between the nurse manager competencies and the demographic and work-related variables (such as age and years of work experience). The regression models were controlled for these personal and work-related variables. The presence of multicollinearity was excluded by examining the correlations among the predictor variables (*r* < 0.60), tolerance (ranging from 0.130 to 0.216), the variance inflation factor (ranging from 4.63 to 9.74), and the condition index (ranging from 1.00 to 8.53).

The overall regression was statistically significant in predicting the job satisfaction (R^2^ = 0.41, F = 159.82, *p* < 0.000) and turnover intention (R^2^ = 0.34, F = 176.32, *p* < 0.000) of nurses. Regression analyses identified “Team Communication and Collaboration”, “Staff Advocacy and Development”, and “Quality Monitoring and Pursuance” as significant predictors of nurses’ job satisfaction and “Staff Advocacy and Development” and “Team Communication and Collaboration” as significant predictors of nurses’ turnover intention (Table 5).

## 5. Discussion

The aim of this study was to examine the associations between nurse manager competencies and nurse outcomes. The findings were consistent with those of previous studies, upholding the role of nurse managers in stabilizing the nursing workforce in the face of the COVID-19 pandemic [12,30]. The findings supplemented the extant literature by providing empirical support for the specific domains of nurse manager competencies that predict the job satisfaction and turnover intention of staff nurses.

### 5.1. Discussion of the Findings

Regarding nurse outcomes, the findings of this study revealed that one-third of respondents were considering leaving their current workplace; the figure was comparable to or slightly better than those of previous studies, which reported that about 40 to 50 per cent of staff nurses intended to leave their current workplace in Taiwan and the United States [19,20,31]. This can be attributed to two factors. The first is that Hong Kong nurses had previously experienced a similar epidemic situation, which was the outbreak of the severe acute respiratory syndrome (SARS) in 2003; local nurses were more prepared for crises and might not have found the COVID-19 pandemic as distressing as nurses in other countries [32]. The second is that local nurses were noted to have gained a strong sense of professionalism from the public recognition that they received, which could have helped to retain them in the workplace [33].

Staff nurses in this study reported a more positive perception towards three domains of nurse manager competency, including “Change and Resource Management”, “Personal Mastery”, and “Quality Monitoring and Pursuance”. “Change and Resource Management” and “Personal Mastery” were not identified as significant factors predicting the job satisfaction and turnover intention of staff nurses in this study. Past relevant studies conceptualized these areas of nurse manager competency as “Staffing and Resource Adequacy” and “Nurse Manager Ability”, and conflicting findings were noted regarding their predictive effects on nurse outcomes [34,35]. Previous research conceptualized “Quality Monitoring and Pursuance” as “Nursing Foundations for Quality of Care” and “Patient Safety Culture”, and similarly, no conclusive findings were yielded regarding their predictive effectives on nurse outcomes [28,36]. Further studies are required to examine the associations between these specific domains of nurse manager competency and nurse outcomes. The pandemic turned hospitals into battlefields, with nurse managers heavily engaged in effecting changes and directing staff to work according to standards to ensure quality of care [6]. During the period of crisis, intertwined challenges related to resource allocation and change and quality management arose [37]. Many of these challenges were exacerbated by the pandemic situation; for instance, the lack of hospital beds, manpower, and materials, such as personal protective equipment [38]. These challenges called for changes such as the development of new systems and protocols of care, and new ways of working, which were mostly coordinated by nurse managers [38]. Their role in sustaining the quality of care was rated the most important in past studies, involving personal mastery such as their abilities to handle contingencies and projecting a sense of confidence, calm, control, and security during crises [39,40,41].

Among the five domains of nurse manager competency, staff nurses reported a more negative perception towards “Staff Advocacy and Development” and “Team Communication and Collaboration”. Past relevant studies described these areas of nurse manager competency as “Nurse Participation in Hospital Affairs” and “Collegial Relationships”, respectively. Consistent with previous studies, this study identified these domains of nurse manager competency as the key predictors of nurse outcomes [28,35]. In previous research, nurse managers rated their advocacy skills lowest among their other skills, and many nurse managers indicated that they had not been prepared and trained to advocate for co-workers in the workplace [42,43]. They faced difficulties in advocating for professional autonomy or, more specifically, the inclusion of frontline nurses in decision-making processes [42,43]. Scholars have highlighted the need to uphold professional autonomy at work, as nurses with a poorer sense of professional autonomy tended to be more dissatisfied with their job and to experience burnout [44,45]. Regarding team effectiveness, findings of previous research indicated that communication and collaboration could directly impact nurse outcomes, and these were compromised due to the lack of time and increased workload resulting from the pandemic situation [6,46]. Elements underlying this area of competency were referred to in previous studies as “human skills”, consisting of communication and relationship management skills [40], and as “directive functions”, concerning the ability of nurse managers to effectively communicate and work with teams, be open to suggestions, and respect nurses.

### 5.2. Implications for Policy, Practice, and Education

The findings of this study have implications for the development of competency assessment tools or performance appraisal tools as well as for the designing of competency-based academic and training programs for nurse managers. The *Nurse Manager Competency Scale* captured the areas of competency crucial for managerial success. With further validation, it could be adopted as a tool to assess the competencies of nurse managers against standards of performance [47]. Regarding the training of nurse managers, previous studies have indicated that a one-off education program failed to improve the competencies of nurse managers [37], and many scholars have stressed the importance of providing systematic and continuous training to nurse managers [41,48]. The findings of this study can serve as a basis for designing these training programmes. Because the study was conducted during the COVID-19 pandemic, it has also taken into account the competencies that nurse managers should learn in order to manage crises or health catastrophes such as pandemics [39]. The efforts made by previous researchers to develop training and orientation programs based on nurse manager competencies have proven to be effective in facilitating the role transition of novice nurse managers and in bringing them success [37]. The findings of this study lay a foundation for the development of succession planning programs to identify the right person with the essential competency levels, and to facilitate the role transition of staff nurses to first-line nurse managers. There is also a need to conduct regular educational need assessments, as the competencies required for success may change with time [41,48]. With committed efforts, our future nurse leaders will be better prepared for the challenges ahead, which will ultimately lead to improved staff and organizational outcomes.

Among the various domains of nurse manager competencies, “Team Communication and Collaboration” and “Staff Advocacy and Development” were identified as the key predictors of nurse outcomes. To ensure team effectiveness, the initial effort should be to understand the views and needs of staff nurses, and to develop a clear and transparent chain of command and an open, two-way system of communication with them [6,38]. During periods of crisis and rapid changes, other than face-to-face interactions, nurse leaders may adopt digital platforms such as webinars and virtual meetings to share information and to network and exchange ideas with others during the pandemic [38,49]. Regarding the role of nurse managers in advocating for the interest of staff nurses, it should be noted that staff advocacy cannot be achieved without understanding matters of concern to nurses. Regular, short surveys can be adopted to facilitate nurses to voice their needs and concerns, and these types of advocacy strategies have been largely neglected in management training for nurse managers [38,42,43].

### 5.3. Limitations and Future Research

This study helped to fill the existing gap in the literature on the “critical success factors of nurse managers” (p. 5), which has been regarded by researchers as a growing global priority [12]. However, this study has a few limitations. While the study was cross-sectional and descriptive in nature, the influence of other political and economic factors that might have influenced the variables (e.g., nurse outcomes) under study were not examined. Future researchers can consider adopting longitudinal studies to further examine the causal relationships among multiple variables that have a bearing on nurse outcomes. In addition, the study was subjective in nature, as the measurement was based on the perceptions of staff nurses. Future studies can consider incorporating the use of more objective measures such as actual nurse turnover. The *Nurse Manager Competency Scale* adopted in this study comprised 60 items and was relatively lengthy, and the findings derived from the study were therefore subjected to acquiescence response bias [24]. Lastly, this study was conducted in a single region, and adopted non-random sampling approaches. This led to uncertainty regarding the representativeness of the findings and their applicability to the global context. Future researchers can replicate the study by adopting a more sophisticated sampling approach that allows for unit-level analyses so as to improve the generalizability of the study. The research team faced practical constraints in recruiting nurse managers to the study; further research can therefore be conducted to compare the nurse managers’ perceptions of their own competencies with those evaluated by staff nurses so as to identify the gap in existing management practices.

## 6. Conclusions

The COVID-19 pandemic has brought about many challenges to contemporary healthcare, leading to rapid changes in the provision of care, and to a sense of uncertainly and insecurity among the workforce. Nurse managers play a pivotal role in ensuring both quality of care and workforce stability. This study adds knowledge to the specific domains of competencies that nurse managers require in order to ensure positive nurse outcomes and has implications for what they can put into practice to attain the goal of organizational and workforce stability.

## Figures and Tables

**Table 1:** Psychometric properties of the nurse manager competency scale.

Items	Factor Loading				
	Domain 1: Staff Advocacy and Development	Domain 2: Team Communication and Collaboration	Domain 3: Change and Resource Management	Domain 4: Quality Monitoring and Pursuance	Domain 5: Personal Mastery
1. Understands staff’s unique needs	0.751				
2. Stands up and speaks for colleagues	0.724				
3. Offers prompt assistance to colleagues when needed	0.722				
4. Advocates for the interests of co-workers	0.702				
5. Develops staff potential	0.666				
6. Able to address the needs of staff	0.622				
7. Provides an accurate appraisal of staff performance	0.607				
8. Delegates work according to the individual’s potential	0.589				
9. Provides advice to colleagues on their career development	0.521				
10. Offers learning opportunities to colleagues	0.483				
11. Offers promotion opportunities to colleagues	0.450				
12. Shares with others his/her own expertise	0.448				
13. Respects others’ views		0.775			
14. Is willing to accept others’ opinions		0.723			
15. Strives to listen to colleagues’ voices		0.684			
16. Views issues from others’ perspectives		0.676			
17. Establishes trusting relationships		0.655			
18. Collaborates effectively with colleagues		0.631			
19. Incorporates multiple perspectives when managing incidents at work		0.597			
20. Shows appreciation to others		0.571			
21. Provides communication platforms		0.540			
22. Mediates conflicts among colleagues		0.522			
23. Bridges communication between hospital administrators and frontline workers		0.507			
24. Cultivates team spirit		0.468			
25. Facilitates collaboration with other departments		0.462			
26. Maintains day-to-day communication with colleagues		0.442			
27. Leads the team effectively		0.421			
28. Is flexible enough to make changes			0.689		
29. Handles unexpected incidents efficiently			0.682		
30. Effectively responds to sudden increases in demand for resources			0.660		
31. Effectively responds to sudden increases in demand for manpower			0.649		
32. Is willing to adopt new technological innovations in healthcare			0.635		
33. Keeps abreast with changes in health technology			0.570		
34. Scrutinizes the use of resources during times of change			0.540		
35. Forecasts the resources needed during times of change			0.529		
36. Facilitates changes at work			0.464		
37. Transforms changes into opportunities for advancement			0.415		
38. Directs colleagues towards goal setting				0.665	
39. Incorporates professional values in goal setting				0.635	
40. Pursues quality of care				0.596	
41. Incorporates organizational values in goal setting				0.524	
42. Develops initiatives to promote quality of care				0.514	
43. Proactively identifies risks to safeguard quality of care				0.503	
44. Pursues advancements in nursing				0.499	
45. Monitors staff performance to ensure quality of care				0.487	
46. Cultivates a culture of quality				0.489	
47. Values evidence-based practices				0.446	
48. Direct the team towards achieving quality of care				0.429	
49. Manages his/her own emotions well					0.666
50. Is open-minded					0.662
51. Is caring					0.607
52. Is fair					0.589
53. Is genuine					0.566
54. Is positive					0.551
55. Is knowledgeable about generational diversity					0.503
56. Is organized					0.478
57. Is hard-working and efficient					0.442
58. Is decisive					0.420
59. Is passionate about work					0.404
60. Is clinically knowledgeable and proficient					0.412
**No. of items**	12	15	10	11	12
**Eigen values**	4.22	3.54	2.65	2.13	1.85
**Explained variance (%)**	19.30	14.78	10.64	8.55	5.03
**Cumulative explained variance (%)**	19.30	34.08	44.72	53.27	58.30
**Cronbach’s alpha values**	0.960	0.948	0.924	0.902	0.898

**Table 2:** Demographic and work-related characteristics of the respondents (*N* = 688).

	*n* (%)
**Gender**	
Male	113 (16.4)
Female	575 (83.6)
**Age**	
25 or below	34 (4.9)
26–35	227 (33.0)
36–45	178 (25.9)
46–55	187 (27.2)
56 or above	62 (9.0)
**Rank**	
Enrolled Nurse	87 (12.6)
Registered Nurse	422 (61.3)
Advanced Practice Nurse	179 (26.0)
**Type of hospital**	
Public acute hospital	451 (65.6)
Public sub-acute hospital	126 (18.3)
Private hospital	111 (16.1)
**Type of clinical setting**	
Medical	207 (30.1)
Surgical	114 (16.6)
Obstetrics	41 (6.0)
Psychiatry	75 (10.9)
Accident and Emergency	39 (5.7)
Operating Theatre	68 (9.9)
Out-patient Clinic/Community Nursing Service	59 (8.6)
Others	85 (12.4)
**Years of working as a nurse**	
1 to 3 years	55 (8.0)
4 to 10 years	217 (31.5)
11 to 20 years	159 (23.1)
21 to 30 years	172 (25.0)
>30 years	85 (12.4)

**Table 3:** The level of nurse manager competencies (*N* = 688).

Domains and Items of the Nurse Manager Competency Scale	Mean ± SD
**Domain 1: Staff Advocacy and Development**	
1. Understands staff’s unique needs	2.74 ± 1.178
2. Stands up and speaks for colleagues	2.92 ± 1.148
3. Offers prompt assistance to colleagues when needed	3.16 ± 1.119
4. Advocates for the interests of co-workers	3.00 ± 1.143
5. Develops staff potential	3.03 ± 1.053
6. Able to address the needs of staff	2.90 ± 1.111
7. Provides an accurate appraisal of staff performance	3.08 ± 1.019
8. Delegates work according to the individual’s potential	3.06 ± 1.102
9. Provides advice to colleagues on their career development	3.11 ± 1.027
10. Offers learning opportunities to colleagues	3.23 ± 1.035
11. Offers promotion opportunities to colleagues	3.15 ± 1.027
12. Shares with others his/her own expertise	3.30 ± 1.002
**Domain 2: Team Communication and Collaboration**	
13. Respects others’ views	3.01 ± 1.113
14. Is willing to accept others’ opinions	2.92 ± 1.110
15. Strives to listen to colleagues’ voices	3.00 ± 1.091
16. Views issues from others’ perspectives	2.89 ± 1.121
17. Establishes trusting relationships	2.87 ± 1.137
18. Collaborates effectively with colleagues	3.07 ± 1.075
19. Incorporates multiple perspectives when managing incidents at work	3.16 ± 1.065
20. Shows appreciation to others	3.19 ± 1.015
21. Provides communication platforms	2.88 ± 1.116
22. Mediates conflicts among colleagues	3.00 ± 1.033
23. Bridges communication between hospital administrators and frontline workers	3.07 ± 1.116
24. Cultivates team spirit	3.14 ± 1.114
25. Facilitates collaboration with other departments	3.28 ± 1.052
26. Maintains day-to-day communication with colleagues	3.00 ± 1.067
27. Leads the team effectively	3.11 ± 0.960
**Domain 3: Change and Resource Management**	
28. Is flexible enough to make changes	3.05 ± 1.058
29. Handles unexpected incidents efficiently	3.35 ± 1.085
30. Effectively responds to sudden increases in demand for resources	3.41 ± 1.021
31. Effectively responds to sudden increases in demand for manpower	3.00 ± 1.197
32. Is willing to adopt new technological innovations in healthcare	3.24 ± 0.940
33. Keeps abreast with changes in health technology	3.26 ± 0.966
34. Scrutinizes the use of resources during times of change	3.10 ± 1.004
35. Forecasts the resources needed during times of change	3.09 ± 0.922
36. Facilitates changes at work	3.01 ± 1.071
37. Transforms changes into opportunities for advancement	3.02 ± 1.059
**Domain 4: Quality Monitoring and Pursuance**	
38. Directs colleagues towards goal setting	3.35 ± 0.942
39. Incorporates professional values in goal setting	3.10 ± 0.987
40. Pursues quality of care	3.10 ± 1.070
41. Incorporates organizational values in goal setting	3.14 ± 0.961
42. Develops initiatives to promote quality of care	3.14 ± 1.028
43. Proactively identifies risks to safeguard quality of care	3.03 ± 1.107
44. Pursues advancements in nursing	3.21 ± 1.014
45. Monitors staff performance to ensure quality of care	3.44 ± 0.974
46. Cultivates a culture of quality	3.26 ± 0.960
47. Values evidence-based practices	3.17 ± 0.950
48. Direct the team towards achieving quality of care	3.13 ± 1.034
**Domain 5: Personal Mastery**	
49. Manages his/her own emotions well	3.15 ± 1.171
50. Is open-minded	2.90 ± 1.143
51. Is caring	3.02 ± 1.107
52. Is fair	2.88 ± 1.131
53. Is genuine	3.15 ± 1.089
54. Is positive	3.17 ± 1.076
55. Is knowledgeable about generational diversity	2.98 ± 0.997
56. Is organized	3.13 ± 1.063
57. Is hard-working and efficient	3.17 ± 1.099
58. Is decisive	3.12 ± 1.037
59. Is passionate about work	3.44 ± 0.996
60. Is clinically knowledgeable and proficient	3.49 ± 0.968

**Table 4:** Associations between nurse manager competencies and nurse outcomes.

	Pearson’s *r* *	
	Job Satisfaction	Turnover Intention
**Nurse manager competencies**	0.60	−0.58
**Team Communication and Collaboration**	0.59	−0.57
**Staff Advocacy and Development**	0.59	−0.58
**Change and Resource Management**	0.57	−0.52
**Personal Mastery**	0.58	−0.57
**Quality Monitoring and Pursuance**	0.56	−0.53

* All correlation were significant at *p* < 0.001.

**Table 5:** Predictors of staff nurses’ job satisfaction and turnover intention.

Predictor Variables	Job Satisfaction				Turnover Intention			
	B	β	t	*p*	B	β	t	*p*
**Team Communication and Collaboration**	0.284	0.289	3.035	0.002	−0.223	−0.243	−2.511	0.012
**Staff Advocacy and Development**	0.227	0.229	2.345	0.019	−0.309	−0.347	−3.581	0.000
**Change and Resource Management**	0.116	0.108	1.352	0.177	−0.036	−0.036	−0.424	0.672
**Personal Mastery**	0.142	0.140	1.264	0.207	−0.106	−0.111	−0.946	0.344
**Quality Monitoring and Pursuance**	0.203	0.213	2.164	0.031	−0.027	−0.027	−0.327	0.744

## Data Availability

The data that support the findings of this study are available from the corresponding author upon reasonable request.
